# Reduction of Management Costs and Avoidance of Air Release of Carcinogens Through a Waste Segregation Program in a Brazilian Medical Institution

**DOI:** 10.3389/fpubh.2020.583962

**Published:** 2020-12-07

**Authors:** Fabiane Kellem Oliveira Cesario, Renata Pereira Fontoura, Amarildo Henrique da Conceição Junior, Amanda Gentil Cruz, Nidah Fawzi Said Nimer, Poliana Brito Morais, Brenda Monteiro Omena, Edivaldo Bazilio dos Santos, Anderson Arantes Silvestrini, Rosângela Vieira Andrade

**Affiliations:** ^1^DF Star, Brasília, Brazil; ^2^Genomic Sciences and Biotechnology Department, Catholic University of Brasilia (UCB), Brasília, Brazil; ^3^Clinical Research Department, D'Or Institute for Research and Education, Brasília, Brazil; ^4^Academic League of Oncology of Base Hospital, Brasília, Brazil; ^5^Faculty of Medicine, University of Brasilia (UnB), Brasília, Brazil

**Keywords:** waste management, incineration (INC), hospital waste disposal, toxic particles, gases, dioxins, carcinogenic agents, oncology

## Abstract

Hospital waste management is a current sustainability challenge. Although not always performed, the most applied approach in current protocols is the proper segregation of waste. The incineration of hospital waste is an significant source of emission of specific toxic particles and gases. We highlighted dioxins, whose representatives have been considered carcinogenic agents since 1994. Several experimental and epidemiologic studies have shown greater cancer morbidity and mortality associated with dioxin exposure. In the present study, we presented the impact of a hospital waste management program implemented in an oncology institution based on proper segregation and consequent reduction of incinerated mass. Data were collected for 8 years and the waste was separated into five categories: infectious (A4), chemical (B), recyclable (DR), non-recyclable (DNR), and sharps (E). The classes addressed to incineration were A4, B, and E. A team education starting from the admission process and with a continued education program was essential for a successfully implemented program. We achieved a 66% saving of waste from incineration, equivalent to 76 tons, of which 71.9 tons corresponded to recyclable waste. If the waste separation protocol was not implemented, the biohazardous and chemical material would contaminate the rest of the residues, making incineration as a final destination mandatory for all the waste. This scenario would result in significantly more dioxins release and a 64% higher cost of waste management. This low-cost implementation measure was effective in the cost reduction of waste management and minimization of air release of human carcinogens.

## Introduction

Waste management, which is a subject with increasing interest in the world, is a sustainability challenge for government regulatory institutions. Because the hospitals are significant sources of human waste, many regulatory governmental agencies have established protocols on the management of the residues generated by the health care institutions. Some classes of hospital waste such as the infectious and sharp materials must be incinerated to ensure biosafety effectiveness and avoid biochemical incidents (1, 2).

Hospital waste generates a considerable number of particles and amounts of gases that have been associated with cancer incidence, mortality and morbidity (2–4). In this study, we highlighted the polychlorinated dibenzo-p-dioxins (PCDD), which are commonly called dioxins. Dioxins have been considered as carcinogenic agents since 1994 Many studies described their effect on the mechanism of carcinogenesis, inhibition of the apoptosis via protein kin B (AKT), extracellular signal-regulated protein kinase 1 and 2 (ERK 1/2) and the cytochrome P450 enzyme CYP1A1 (5–12).

The half-life of PCDD is approximately 10 years. It causes a long-term threat to the lives of animals and humans, mainly due to the bioaccumulation effect. There is a higher concentration of toxins at each level of the food chain, being maximum in the human organism (1, 13). Several epidemiological studies with a population exposed to the mixture of dioxins, furans and other chemicals observed an increased incidence of cancer in different organs as well as reproductive effects, immune deficiency, endocrine dysfunctions such as diabetes mellitus; changes in testosterone and thyroid hormone levels, cognitive and behavioral changes in newborns, damages to liver tissue; elevation of lipids and damage to the skin (3, 14, 15).

Also, higher concentrations of serum and urinary PCDD were observed in individuals who lived near incinerators or who consumed food produced near them (2). People living in the neighborhood of the integrated waste management unit compared to cities that do not have operating solid waste incinerators were reported to have a three to four times higher risk of developing cancer (16). There is also an association between living near incinerators and the development of Non-Hodgkin lymphoma, soft tissue sarcoma, colon and liver cancer and increased mortality from other tumors such as stomach, gallbladder, lung and pleura (2, 17–20).

Therefore, actions aiming at the reduction of incinerated material from hospital waste have a crucial role in cancer primary prevention. Regulatory agencies on waste management recommend proper segregation of the waste as the main management strategy, as an intention to prevent recyclable and other non-infectious and non-biohazardous waste to be unnecessarily incinerated (1, 3, 21).

In this study, we aimed to present the effect of a hospital waste management program implemented in an oncology care institution composed of different facilities over the city. Additionally, we discussed the relevance of this strategy in public health and focused on the prevention of malignant neoplasms associated with dioxin exposure.

## Methods

The program described herein happened in an institution specialized in oncology in the city of Brasília–Federal District, Brazil. Our institution was composed of eight different facilities in different addresses across the city. Our waste management program was implemented in December 2009 and followed the current Brazilian legislation (21). The implementation went through the stages of planning, review and approval. The team training and main step of the program were conducted by one manager. This person elaborated a lecture regarding the types of waste generated at the institution, standard identification of dumpsters and bags, appropriate disposal based on the types of waste; using detailed examples, risks of incorrect disposal and final destination and treatment.

This lecture was provided to every new personnel admitted to our institution and repeated every 6 months. The attendance of each employee on this training was obligatory and controlled by the Human Resources Department. The Infection Control Program included waste segregation actions in its regular quality audits to ensure the effectiveness of the program. Additionally, in case of an incidental perforation due to incorrect discharging, the program manager received a notification and had to analyze the event. Once identified the area where it happened, he performed another team training focusing on the mistakes that led to the accident.

Another factor included in the program was the data collection. Since our institution was composed of different facilities, each unit was responsible for its data collection. In each unit, the professionals weighed the waste per class and recorded the weight on a control table daily. This table was typed weekly in electronic form and the monthly control was kept under the custody of the institution's general supervisor of waste management.

The waste was separated into five categories: infectious (A4), chemical (B), recyclable (DR), non-recyclable (DNR), and sharps (E). The classes addressed to incineration were A4, B, and E (21). The Burning Report from the plant responsible for the incineration of our waste was also according to the current Brazilian legislation (21). The incinerator used in this study had a rotary kiln with a processing capacity of 700 kg/h of waste, located in Anápolis, Goiás, Brazil. The burning of the waste happened after the mixing process gathering the classes A, B, and E utilizing the great caloric potential of this merger. Thus, it was possible to increase the temperature and consequently to achieve more efficient combustion of waste. After the incineration, ashes classified as inert waste were liable to be reused/recycled, for example, in civil construction, as agricultural inputs, ecological bricks, asphalt plants or concrete artifacts.

### Data Collection

Data collection happened from 2010 to 2017 at five outpatient services (facilities 1–4 and 6) and a pharmacy unit (facility 5) A general manager computed the data forming monthly reports. Facilities 1, 2, 3, 4, and 6 are clinics focused in the follow-up and treatment of cancer patients, that offer outpatient treatment regimens including intravenous (IV) infusion of chemotherapy, monoclonal antibodies, antibiotics, antifungals, iron replacement therapy and pulse therapy. Facilities 1, 2, 3, 4, and 6 also perform procedures like myelogram, bone marrow biopsy, heparinization of totally implanted central venous catheters and lumbar puncture. Facilities 1 and 2 were founded in 2010, while facilities 3 and 4 were founded in 2012 and facility 6 was founded in 2015. Facility 5 is a pharmacy unit that focuses on handling and distribution of drugs. Facility 5 was founded in 2012.

### Statistical Analysis

For statistical analysis, we divided the waste products into two groups: incinerated (A4, B e E) and non-incinerated (R and NR). Data were presented as the amount of waste produced in kilograms (kg). Linear regression was used to estimate the residues from the incinerated and non-incinerated groups in each facility, considering time as an explanatory variable. A moving averages filter (data processing) with a 6 months lag, in which each point on the graph presents the average of the values observed in the last 6 months, was also applied to reduce statistical noise in the series. Results were presented as an estimation and standard variation. One-way ANOVA was used to compare the differences between the groups. Significance was set at *p* < 0.05. The trend was obtained from the additive decomposition of the series using moving averages. We applied the Augmented Dicke-Fuller Test to verify the stationarity of the series which we considered to be stationary if *p*-value was < 0.05. All analyses were performed using the R software.

## Results

In 8 years, the waste production was equivalent to 6,048.08 kg of infectious material (A4); 23,115.10 kg of chemical waste (B); 71922.25 kg of recyclables (R); 4531.36 kg of non-recyclables (NR); and 10541.87 kg of sharps (E) (Table 1). Of the total waste generated, 66% did not undergo incineration, equivalent to 76 tons, of which 71.9 tons was recyclable.

**Table 1:** Amount of waste produced per year, divided into categories.

**Year**	**A4**	**B**	**D**		**E**	**Total**
			**R**	**NR**		
2010	1463,19	1882,59	5391,82	169,76	1007,60	**9914,959**
2011	962,42	2211,76	6279,35	420,19	1086,55	**10960,267**
2012	599,84	2130,21	6585,81	266,86	1057,45	**10640,163**
2013	442,17	3054,44	9479,40	512,05	1460,50	**14948,561**
2014	539,04	2781,69	10453,83	674,14	1430,79	**15879,486**
2015	581,90	3111,91	10770,33	943,25	1503,80	**16911,181**
2016	651,53	3717,11	10732,23	914,89	1458,96	**17474,730**
2017	808,00	4225,38	12229,49	630,23	1536,22	**19429,313**
**Total (kg)**	**6048,08**	**23115,10**	**71922,25**	**4531,36**	**10541,87**	**116158,660**
**Total (%)**	**5%**	**20%**	**62%**	**4%**	**9%**	**100%**

*A4, infectious waste; B, chemical waste; D, common garbage; R, common recyclable waste; NR, common non-recyclable waste; E, sharps. Colors and Bold characters were adopted just to highlight the “Total” values*.

**Figure 1:**
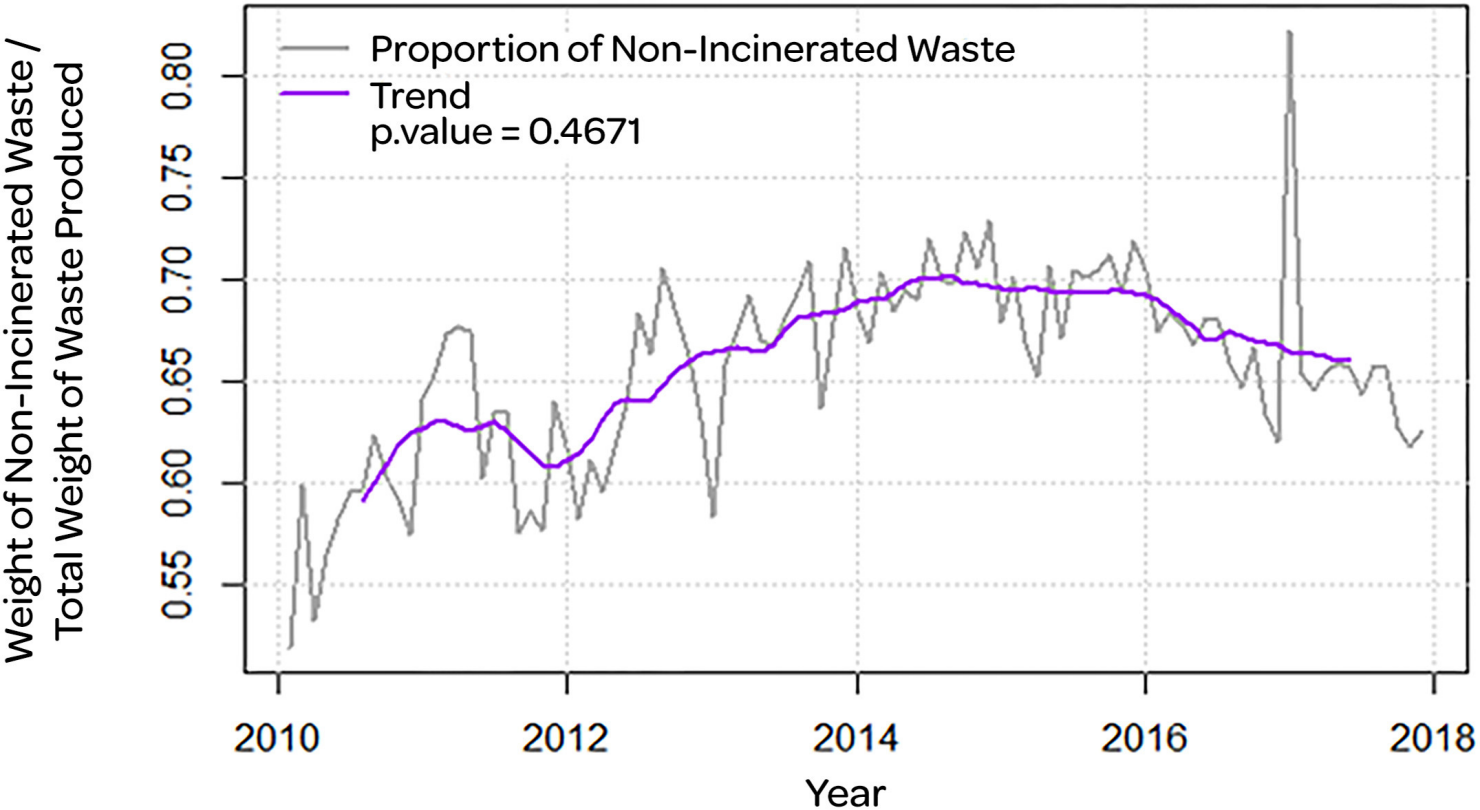
Proportion of total non-incinerated waste (gray line) with trend (purple line) and *p*-value of the stationarity test.

Figure 1 showed the proportion of total non-incinerated waste over the total amount of waste produced. We can see that most of the time the amount of non-incinerated waste remained above 60%. The Augmented Dicke-Fuller Test found that the series was not stationary (*p* = 0.4671), which indicated the presence of a trend. Additionally, the proportion grew until 2015 and there has been a slight decline ever since.

Table 2 showed the results of linear regressions for waste groups in each facility. Such regressions emphasized that time (in months) was an explanatory variable for the amount of waste produced. In facilities 1, 3, and 6 there was an increase of 13,208, 13,181, and 53,840 kg on average, respectively, of non-incinerated waste per month. This increase was significant at a level of 5%. Facility 3 also showed a significant increase in the total waste of 15,888 kg on average per month. Facility 6 significantly increased the incinerated waste (36,030 kg on average per month) and even faster the total waste (89,870 kg on average per month). Facility 2 had a significant decrease in the incinerated and non-incinerated waste and total.

**Table 2:** Estimates of the change in the amount of incinerated and non-incinerated waste (kg) produced in each facility of the medical facility per month and *p*-value of the stationarity test.

**Facility**	**Waste type**	**Estimate**	**Standard deviation**	***p*-value**
Facility 1	Incinerated	−2.241	3.859	0.5640
	Non-Incinerated	**13.208**	**5.492**	**0.0195**
	Total	10.966	8.824	0.2190
Facility 2	Incinerated	–**21.983**	**3.959**	**<0.0001**
	Non-Incinerated	–**43.457**	**5.907**	**<0.0001**
	Total	–**65.440**	**9.204**	**<0.0001**
Facility 3	Incinerated	2.707	2.820	0.3410
	Non-Incinerated	**13.181**	**4.355**	**0.0037**
	Total	**15.888**	**7.053**	**0.0281**
Facility 4	Incinerated	1.495	1.170	0.2060
	Non-Incinerated	6.542	4.348	0.1370
	Total	8.037	5.184	0.1260
Facility 5	Incinerated	5.057	2.909	0.0870
	Non-Incinerated	−4.256	5.574	0.4480
	Total	0.802	7.446	0.9150
Facility 6	Incinerated	**36.030**	**10.130**	**0.0011**
	Non-Incinerated	**53.840**	**22.170**	**0.0206**
	Total	**89.870**	**23.750**	**0.0006**
Total	Incinerated	**45.928**	**3.481**	**<0.0001**
	Non-Incinerated	**111.000**	**7.388**	**<0.0001**
	Total	**156.900**	**9.855**	**<0.0001**
Total Cost*	Segregation scenario	**137.78**	**10.44**	**<0.0001**
	No segregation scenario	**470.78**	**29.56**	**<0.0001**

*Total waste management cost is calculated in the real observed scenario in which segregation was applied and a simulated scenario with no segregation*.

* *Costs are expressed in brazilian currency (R$). The line with a p-value < 0.05 are presented in bold characters. Colors were adopted just to highlight the “Total” values*.

Altogether, the facilities had a significant increase in incinerated, non-incinerated, and total waste. However, if the segregation measure has not been adopted, the increment of incinerated waste would be faster. All the facilities kept a proportion of non-incinerated garbage above 60% most of the time. The facilities 1, 2, 3 and 5 did not show significant stationarity in the Dickey-Fuller test. There was a great loss of data on Facility 2, which can damage the test result and make it inconclusive.

**Figure 2:**
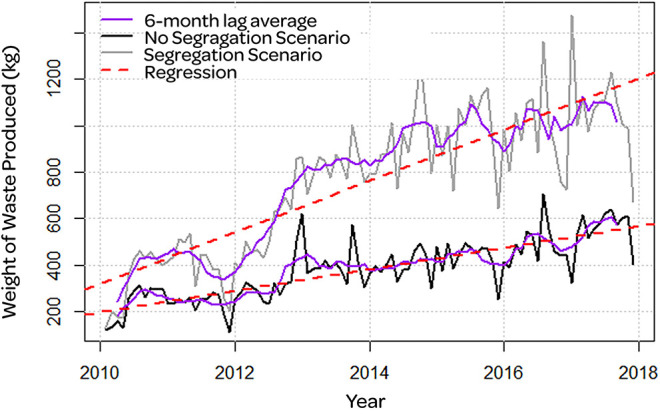
Amount of waste produced that was not incinerated (gray line) and which was incinerated (black line). Moving averages filter with 6 months lag (purple line) and regression line (red dashed line).

**Figure 3:**
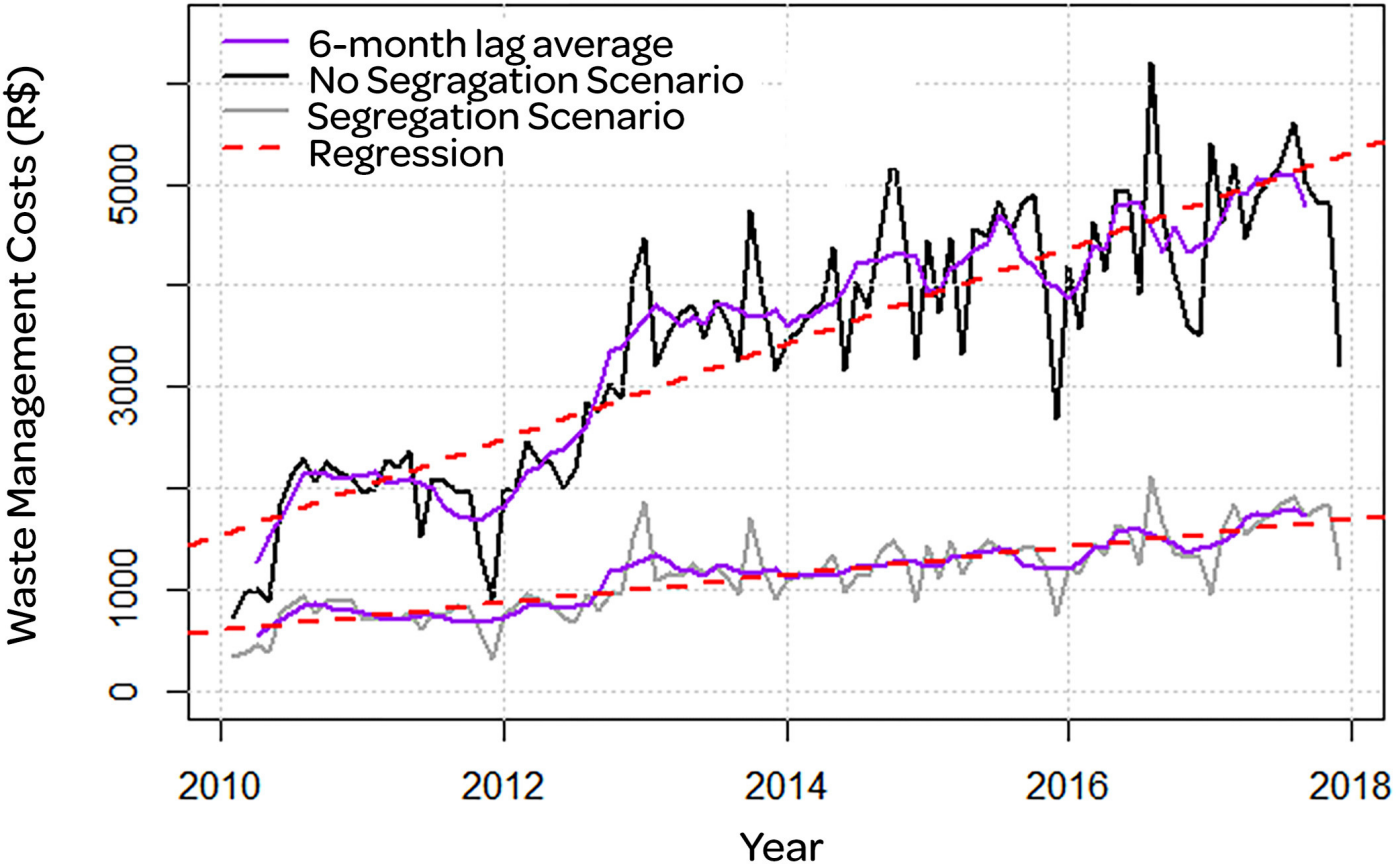
Costs associated with incineration in the scenario with waste segregation (gray line) and in the scenario without segregation (black line). Moving averages filter with 6 months lag (purple line) and regression line (red dashed line). Costs are expressed in brazilian currency (R$).

Figure 2 showed the series over the years for the total amount of waste produced (Figure 3) presents the costs of waste management. We identified non-incinerated waste in the gray and black incinerated waste. The purple line represented the filter through the method of moving averages with a 6-month lag, which helped us to identify cycles and trends of the series. We identified the regression line in red. Also, we did not identify a seasonality trend in our waste production.

Finally, there was a significant increase in costs of R$ 137.78 on average per month in the scenario with segregation. The amount paid for incineration was R$ 3,000.00 per ton, which was R$ 119,115.14 in total after 8 years. We did a simulation of costs in a non-segregation scenario considering that without segregation all waste produced needed to be regarded as potentially infectious, and hence, incinerated. In the non-segregation plot, the costs would increase to R$ 470.78 on average per month, more than three times higher than the monthly increase of costs in the segregation scenario. The total cost after 8 years in a non-segregation scenario would be R$ 348,475.98, more than 190% higher than the total cost in the segregation scenario. The differences between these scenarios, considering aggregate costs, were presented in Figure 3. According to Brazilian legislation, the facilities considered to be major waste generators started to be responsible for the handling of non-incinerated waste (classes NR and R) only in 2019. Thus, the government urban cleaning service was responsible for the management of non-incinerated waste produced by our institution, adding no further expense.

## Discussion

The generation of waste is an inevitable result of patient care. In our experience, there was an increase in waste production over time, proportional to the volume of care provided by the institution. As expected, we perceived a gradual increase in chemical waste generation, which was directly associated with the advancement of oncology care and hence a larger number of chemotherapy protocols over time, which may explain the increase in incinerated waste even in the segregation scenario.

The particular concern about the management of hospital waste is the mandatory incineration of classes A, B, and E due to their contaminating and harmful properties. The main group of dioxins released by incineration, PCDDs, has been associated with the development of various malignancies (2, 16–20).

On the other hand, the non-infectious waste produced in hospitals can be sent to recycling programs and municipal waste management and reducing the amount of incinerated material and the cost of waste treatment through the segregation programs (1). Therefore, the segregation of waste, reserving incineration only to the classes for which it is imperative, is the most appropriate strategy to reduce the impact of hospital waste.

Many regulatory agencies elaborate legislations and orientations on how to properly segregate and discard the residues. Despite that, there are many barriers to the success of this strategy (1). We highlighted, through our experience, the non-standardization and inadequate training of hospital teams which was the strongest point of our waste management program. The staff training, starting at the admission and with a continued education program, providing knowledge on the importance of segregation and clarifying the identifications of the disposal containers was essential to the success of the implemented program.

The continuous data collection and supervision, conjoined with the Human Resources and Infection Control Departments allowed the internal feedback to evaluate the success of the efforts led by the segregation managers. We observed a reduction in the amount of infectious material (A4) over the years, meaning that the allocation of materials in containers for this class has been increasingly reserved for only infectious materials, increasingly over time. In a scenario of increasing the total amount of waste produced, this denoted the achievement of the appropriate team training.

Our program allowed 62.6% of the total waste produced to be separated and destined for safe recycling. Besides, the incineration technique adopted allowed the resulting ashes to be inert and therefore reused in other contexts. It was possible to obtain the incineration of only 34% of the total waste produced, surpassing the efficiency of waste stratification strategies previously described in the consulted literature with rates around 42% (22, 23).

In addition to the release of toxins, incineration is related to a substantial increase in the cost of waste management. As shown in Figure 3, proper waste management with careful segregation allowed a spare of 65.85% of the cost, corresponding to R$ 229,360.84 and corroborating with the data provided by other authors (1).

A major advantage of our program is the multi-professional approach. The mutual collaboration between the teams of quality control, human resources and infection control, and the optimized utilization of the workforce allowed that all the efforts regarding team training, data processing and reevaluation of results could be performed with no extra personnel hiring.

Our services already owned appropriate waste disposal containers before our program began, even though the team had never been trained in the to perform the segregation. This allowed us to initiate our program without any initial investment. If a service that does not own appropriate waste disposal containers wishes to apply a segregation program that is similar to ours, it would need to acquire both definitive containers (that may be utilized for the disposal of DR and DNR type waste), disposal bags (that may be utilizes for the disposal of A and B type waste) and disposable containers (that may be utilized for the disposal of E type waste). The total cost of this initial investment may vary from country to country but are definitively lower than the economy of R$ 229,360.84, observed in our service. Since the strategy did not include the admission of new staff or the purchase of new administration tools, the exact costs of our program cannot be defined. Our program consisted of attributing new functions to the regular staff and did not demand a considerable amount of extra work time. Thus, considering the savings on the costs of waste management, it was cost-effective.

More detailed data of our results are given in Table 2. Altogether, the facilities had a significant increase in incinerated, non-incinerated and total waste. However, the increment of incinerated waste would be faster if the segregation measure was not adopted. We observed that all the facilities kept a proportion of non-incinerated garbage above 60% most of the time. Facility 2 had a significant decrease in incinerated, non-incinerated waste and total. We denote that there was a great loss of data on Facility 2, which can damage the test result and make it inconclusive. The only facility that registered a significant increase in the amount of incinerated waste over the months was number six. It was also our unit with the highest proportion of B type waste (23, 47%), which can be attributed to the fact that it was the facility with the greatest expansion in the amount of care provided. We observed a peak in the proportion of non-incinerated waste in the 1st months of 2017. We investigated possible causes of this finding and regarded the patient volume variations, inauguration or closing of centers of treatment. However, none of these factors had a close temporal relationship with the observed peak. Consequently, we could not explain whether the observed peak had an identifiable cause or just resulted from data noise.

Since the data collection was a part of our waste management program, it only started after its implementation. Therefore, this study could not compare our data with the already existing data on waste production before the program started. We observed growth in the proportion of non-incinerated waste through time and did not find significant stationarity in this matter. Hence, we can conclude that the proportion of incinerated waste decreased. The analysis of the stationarity of the proportion of incinerated waste could supply the absence of data from before the implementation of the program.

Strict segregation is associated with a reduction of 99.5% of dioxins released into the atmosphere, in addition to other contaminants and particulates (24). The total amount of 76,453.61 kg of residues was spared from incineration through the efforts of our program, corresponding to 66% of our total waste production.

One of the limitations of our study was that it was not possible to establish the correct number of dioxins that were not released. This could be because that different amounts of gas are released after the incineration of each type of material. So, we would have to analyze the gases produced after every incineration to obtain such data.

## Conclusion

To conclude, a well-planned waste management program can have a major effect on reducing the amount of incinerated waste and waste management costs. Also, we estimate that the significantly reduced number of residues incinerated resulted in a significant reduction in the total amount of carcinogens released. Thereby, the population who live around the plant we utilized might have a reduced and smaller risk of cancer development and further studies should be conducted to corroborate this hypothesis.

## Data Availability Statement

The raw data supporting the conclusions of this article will be made available by the authors, without undue reservation.

## Author Contributions

The study was designed by FC. The data was collected by ES. The literature review and discussion were done by ACr, NN, PM, and BO. Data analysis and result compilation were done by RF and ACo. All authors discussed the results and contributed to the final manuscript.

## Conflict of Interest

The authors declare that the research was conducted in the absence of any commercial or financial relationships that could be construed as a potential conflict of interest.
